# Airway brushing as a new experimental methodology to detect airway gene expression signatures in mouse lung squamous cell carcinoma

**DOI:** 10.1038/s41598-018-26902-7

**Published:** 2018-06-11

**Authors:** Jing Pan, Donghai Xiong, Qi Zhang, Eva Szabo, Mark Steven Miller, Ronald A. Lubet, Yian Wang, Ming You

**Affiliations:** 1 Cancer Center, Medical College of Wisconsin, 8701 Watertown Plank Road, Milwaukee, WI 53226 USA; 2 Department of Pharmacology & Toxicology, Medical College of Wisconsin, 8701 Watertown Plank Road, Milwaukee, WI 53226 USA; 3 Chemopreventive Agent Development Research Group, Division of Cancer Prevention, National Cancer Institute, 9609 Medical Center Drive, Rockville, MD 20850 USA

## Abstract

As a consequence of exposure to environmental toxicants, a “field cancerization” effect occurs in the lung, resulting in the development of a field of initiated, but morphologically normal appearing cells within a damaged epithelium containing mutations in oncogene or tumor suppressor genes. Unlike humans, whose airway field of injury associated with lung cancer has long been investigated with airway brushings obtained via bronchoscopy, no methods are available for similar studies in the mouse due to the small size of the murine airways. In this protocol, we describe a detailed method for performing airway brushing from a live mouse, which enables repeated sampling from the same mouse and thus, mimicking the bronchoscopy protocol used in humans. Using this approach in the *N*-nitroso-tris-chloroethylurea (NTCU)-induced mouse lung squamous cell carcinoma (SCC) model, we isolated airway epithelial cells with intact cell membrane structure and then performed transcriptome sequencing (RNA-Seq). We found activation of the PI3K signaling network to be the most significant in cytologically normal bronchial airway epithelial cells of mice with preneoplastic lung SCC lesions. Prolonged exposure to NTCU also induced activation of NF-kappaB (NFƙB), the downstream pathway of PI3K; this NTCU-induced lung SCC progression can be reversed by blocking the NFƙB pathway.

## Introduction

Lung cancer is the leading causes of cancer death worldwide. Of the two main histopathologic classifications, non-small cell lung cancer (NSCLC) and small cell lung cancer (SCLC), approximately 85% of lung cancer falls into the NSCLC category and can be further subclassified as adenocarcinoma, squamous cell carcinoma (SCC), and large cell carcinoma. The histologic classifications oversimplify the heterogeneity and molecular complexity of NSCLC. Therefore, the identification of specific molecular changes in the cancer cells has significant implications, not only for our understanding of lung cancer biology, but also for lung cancer therapies^1^.

Cigarette smoking is the top risk factor for lung cancer, especially for lung SCC. Prolonged exposure to cigarette smoke or other carcinogens creates a field of injury in the cytologically normal epithelium that lines the respiratory tract, reflecting processes associated with the precancerous disease state^2^, such as mutations in oncogenes or tumor suppressor genes induced by inhaled environmental toxicants^3–5^. Molecular characterization of this field of injury in the upper airways can provide novel insights into lung carcinogenesis and identify biomarkers to guide early detection of, and early intervention in, lung cancer^2^. Tissue samples from this extended injured area can be collected in a less invasive manner by bronchial brushing, and have been used to develop a gene expression–based biomarker signature for distinguishing lung cancer in smokers^6^. A gene expression classifier from bronchial airway was also developed to improve the diagnosis of lung cancer^7^.

Murine models for the study of lung cancer have historically been the backbone of preliminary preclinical data to support early human clinical trials, and are critical in advancing our understanding of malignancies and testing therapeutic options before initiating clinical trials^8^. However, due to the small size of the mouse airway that precludes repeated sampling, most investigators have had to euthanize the mice in order to obtain biological samples for testing. This approach requires comparisons of results obtained between different animals when analyzing important molecular events during lung carcinogenesis as well as response to therapy, and thus, introduces unavoidable genetic noise. We, therefore, wished to develop a method to enable comparison of molecular changes from the same mouse and build a more robust model that can be directly compared to studies conducted with human patient samples.

In this study, we developed a non-lethal method of collecting airway epithelial cells from mice and conducted RNA-sequencing analysis using bronchial airway samples from a NTCU-induced lung SCC model to test the relevance of this method against data obtained from human datasets. We identified significant activation of oncogenic pathways, especially the PI3K/Akt pathway, as early events during murine lung SCC development. We further validated these RNA-seq data with NFƙB reporter mice and found that prolonged NTCU exposure induced significant NFƙB activation *in vivo*. Intervention of these important pathways with small molecule inhibitors or genetic modulation with IƙBα super-repressor is sufficient in preventing lung SCC progression.

## Experimental Design

### Anesthesia

We used a ketamine/xylazine cocktail to anesthetize the mice, which is critical for the airway brushing procedure. Ketamine and xylazine are both pharmaceutical grade anesthesia agents. At the recommended doses, this drug combination normally provides 20–30 min duration of anesthesia, which should be sufficient to complete the procedure. Mice administered too much ketamine/xylazine are more likely to stop breathing during the airway brushing procedure, and recover poorly from the anesthesia. Conversely, mice administered too little ketamine/xylazine may struggle during the invasive brushing procedure. If this occurs, more ketamine/xylazine could be given before continuing with the procedure. We found that mice have different sensitivities to this anesthesia cocktail; some mice will not be anesthetized deeply enough even if given extra doses of the cocktail. Therefore, extra mice should be prepared in the event of unanticipated complications. Following the procedure, mice will be placed in a warm, clean, dry, new cage for recovery, and will be returned to the animal facility once they can maintain an upright posture and walk normally about the cage.

### Mice

Mice between 8 and 30 weeks of age are used for airway brushing. Mice of this age are old enough to recover from the anesthetic, the airway size is about 1 mm, therefore, they can adapt to the brushing procedure with less likelihood of fatal complications during the procedure. All studies on animals were approved by the Medical College of Wisconsin Institutional Animal Care and Use Committee (IACUC). All methods were performed in accordance with the guidelines and regulations of IACUC.

## Materials and Reagents

- Ketamine (Avertin, Sigma Aldrich T48402)
- Xylazine (Tert-amyl alcohol, Sigma Aldrich 152463)
- Phosphate Buffered Saline (PBS)
- RLT (Qiagen, 79216)
- 2-Mercaptoethanol (Sigma, M3701)

## Equipment

- Scale (Ohaus, Cat. #HH120D)
- Needles (30 gauge, ½ inch, Becton Dickinson, Cat. #305106)
- Syringes (1 ml, Becton Dickinson, cat. #309602)
- Micro dissecting forceps (George Tiemann Co., Cat. # 160-18)
- Tissue forceps (George Tiemann Co., Cat. #160-16)
- Cytological brush (ConMed, Cat. #133)
- Rodent Intubation Stand (Braintree, Cat. #RIS 100)
- Fiber-Lite Illuminator (Dolan-Jenner Industries, Inc., Model 3100-1)
- Heating pad (Stryker Gaymar, Cat. # TP700)

## Reagent Set-up

### Ketamine/Xylazine Working Solution (10 mg/ml–1 mg/ml)

Add 1 ml ketamine stock (100 mg/ml) and 0.5 ml of xylazine stock (20 mg/ml) to a sterile amber vial with 8.5 ml of PBS. Working solution is stable at room temperature (18–25 °C) for approximately one month. Discard the solution before expiration date.

#### Critical step

Negative pressure amber bottle should be used to avoid contamination.

## Equipment Set-up

Anesthesia recovery cage: place an empty, clean cage on top of heating pad, which was set up at 38 °C.

### Rodent intubation stand and light source

**Figure 1:**
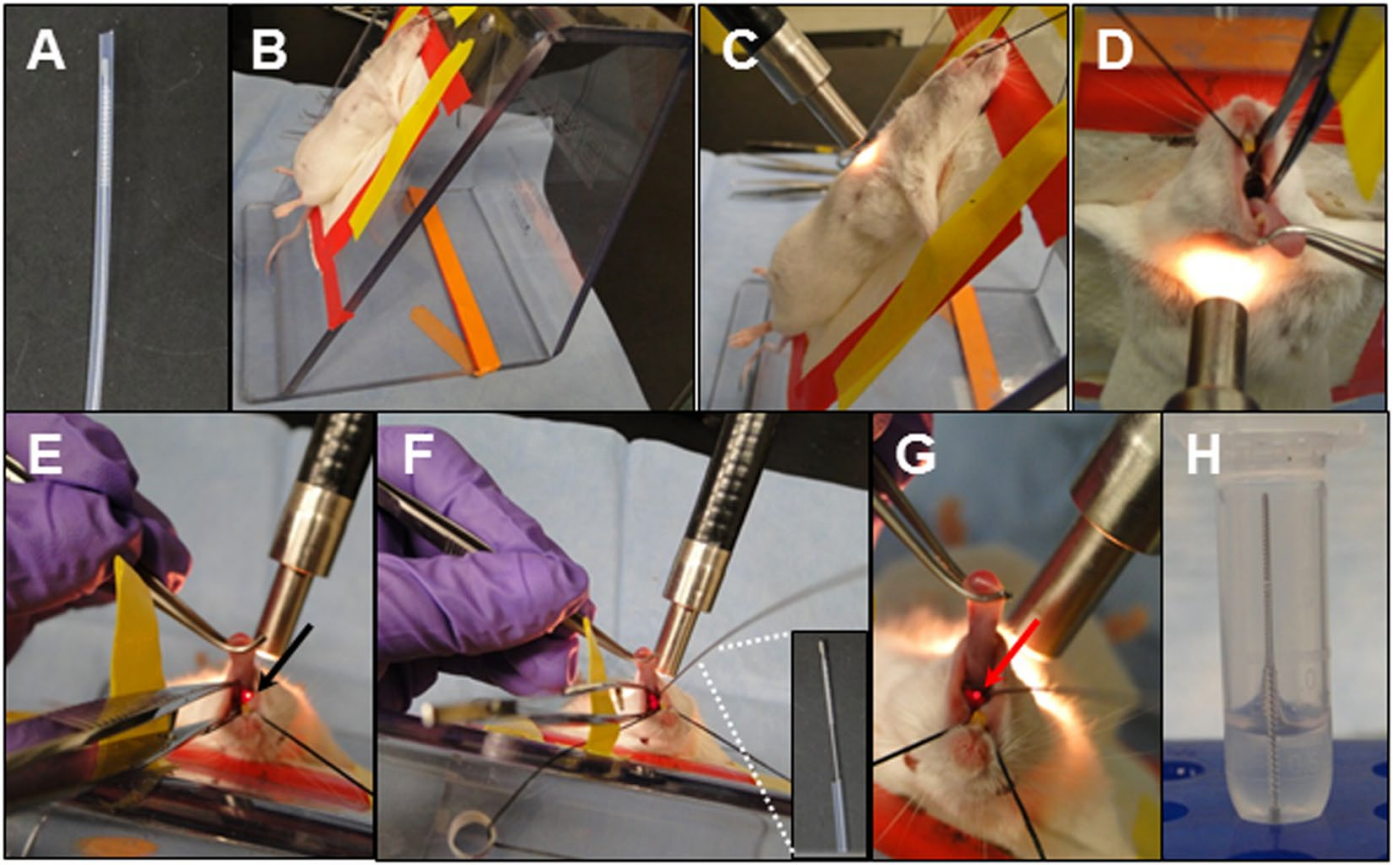
Airway cytological brushing procedure. Cytological brush in sheath (**A**), Anesthetized mice are placed on the stand by their front teeth (**B**), The light is directed on the mouse’s upper chest (**C**), the tongue is gently pulled out using the curved micro dissection forceps, mouth is opened using the tissue forceps(**D**), vocal cord is located by the white light emitted from the trachea (**E**), cytological brush is slid into the trachea (**F**), and the localization of the brush is confirmed by the appearance of the brush handle coming out of trachea (**G**), the brush is then placed in the tube with excess handle cut off (**H**).

For airway brushing, set up the stand and fiber light source on a flat surface as shown in Fig. 1. Prepare cytological brush by cutting the excess length of wire and leave about 20 cm length of the wire, keeping the brush inside of the sheath.

## Procedure

### Cytological brush preparation TIMING ~2–10 min depending on the number of brushes needed for the experiment

1. Cytological brushes come in an individual sterile pack, each has a 1 m long handle which was designed for human use but is too long for mice. The cytological brush handle needs to be cut off to leave a 20 cm long brush with all brushes protected in their own sheath until use (Fig. 1A).

### Animal preparation TIMING ~5–10 min

2. Ketamine/Xylazine administration TIMING ~1–5 min per mouse

Anesthetize mice by intra-peritoneal injection of ketamine/xylazine mixture (0.2 ml for body weight of 20 g). Confirm the mice are fully anesthetized by ensuring that they lack a toe reflex.

#### Critical step

Administering the correct amount of ketamine/xylazine is critical to successfully collecting airway epithelial cells by brushing.

### Visualize trachea opening TIMING ~2-5 min

3. Place mouse on the stand and secure the mouse from its top front teeth with a silk suture; secure mouse forearms with tape (Fig. 1B).

4. Place the Fiber-Lite Illuminator to the mouse’s chest (Fig. 1C).

5. Use the cured micro dissecting forceps in one hand to gently retract the tongue out of the mouth. Use the straight tissue forceps in the other hand to press on the tongue to expand and expose the tracheal opening (Fig. 1D). The straight tissue forceps at this point can rest on the stand with a piece of tape folded up to block its movement (Fig. 1E, yellow tape), and one can use that hand to adjust the Fiber-Lite slightly around the chest until the vocal cords are clearly visualized (Fig. 1E, black arrow).

6. Hold the 1 mm cytological brush parallel with the mouse. Using the bottom front teeth as the supporting point for the brush, directly insert the brush between the vocal cords into the airway (Fig. 1F and inlet).

7. Gently slide the brush down the airway; once it reaches the trachea-bronchial junction, retract the brush gently out of the airway. Localization of the brush inside the airway can also be confirmed by direct visualizing the end of the brush handle coming out of the airway (Fig. 1G),

8. Immediately place the brush into PBS buffer or in RLT and cut off the excess handle. Leave the brush in the buffer (Fig. 1H) and briefly pulse the sample on a vortex mixer to release all cells in solution, which can be used for subsequent cytological analysis or RNA extraction, etc.

#### Troubleshooting

i. When the brush gets into the mouth, avoid contact with the upper jaw or tongue, which will cause oral squamous cells to attach to the brush. In this case, switch to a new brush and start over. The brush sheath can be used to protect the brush from contacting oral squamous cells; however, it may block the view of the tracheal opening.
ii. The proper placement of the brush in the trachea can be confirmed by visualizing the end of the brush clearly located in between the vocal cords (Fig. 1G).

#### Critical Step

iii. The brush needs to stay in its sheath to avoid picking up any oral squamous cells.
iv. Insertion of the brush in the trachea will block the airway and prevent breathing; remove the brush quickly but gently to allow the mouse to breathe.

### Animal Recovery TIMING ~10–30 min

9. Release the mouse from the stand and transfer it to the warm recovery cage on the heating pad to recover. Once it is fully conscious and can walk freely in the recovery cage, it can be returned to its original cage.

## Results

### Highly enriched lung epithelial cells obtained from airway brushing samples

**Figure 2:**
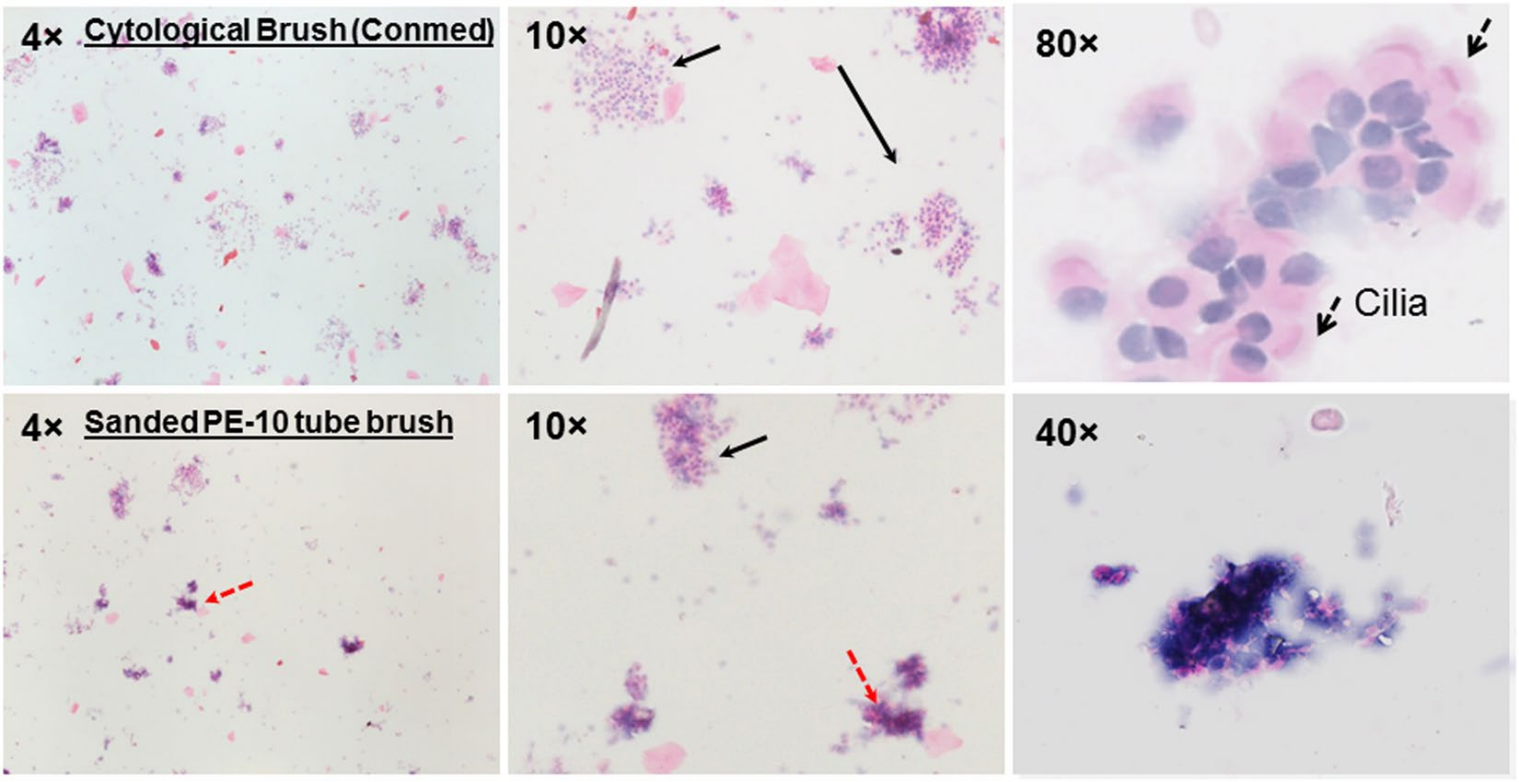
Morphological examination of cells collected from two different brushes. Dotted red arrow: epithelial cells with damaged cell membrane; solid black arrow: epithelial cells with intact cell membrane.

To confirm the cell types obtained from airway brushing methods, we compared cells harvested from airway brushing using a previously reported homemade^9^ brush to a commercially available brush (cytological brush, ConMed, Cat. #133). As shown in Fig. 2, the homemade brush, which uses sanded PE-10 tube^9^, was also able to collect airway epithelial cells. However, the homemade brush caused significant amount of the cells to lose their intact cell membrane structure, leading to a harvest of damaged cells (Fig. 2). In comparison, when a commercially available cytological brush was used, the majority of the cells had intact cell membranes; >90% of the cells were lung epithelial cells as confirmed by H&E staining. One gentle brushing normally collects 50,000~200,000 lung epithelial cells, which is sufficient to produce one high quality slide for IHC or IF staining, or is sufficient to provide ~20 ng of high quality RNA for transcript related studies.

### Airway brushing samples provided high quality RNA for subsequent RNA-seq analysis

To establish proof-of-concept, the trachea-bronchus brushing method was used to collect the lung epithelial cells from two test mice. Total RNA isolation and RNA-seq library preparation was performed on the lung epithelial cells. Qiagen RNeasy® Mini Kit was used for total RNA isolation. Although the amount of sample containing mouse lung epithelial cells collected by brushing was very small, we still successfully extracted approximately 20–30 ng of total RNAs from the tiny brush samples. The integrity of the total RNA samples obtained was very high, with the RIN (RNA integrity number) value being greater than 8, as shown in Fig. S1. The RNA sample quality was analyzed by the widely used Agilent RNA 6000 Pico Chip analysis.

Next, we used the NEBNext Ultra RNA Library Prep Kit from Illumina to construct the RNA-seq libraries for the total RNA samples extracted from the mouse airway brush samples. NEBNext Ultra RNA Library Prep Kit can be successfully used to process the small amounts of starting total RNA samples (10 ng-1 μg). Two RNA-seq libraries were prepared based on these two total RNA samples. The results were excellent, as shown in Fig. S2 (RNA-seq library for samples PJ-1 and PJ-2). The Agilent High Sensitivity DNA Chip analysis showed a narrow distribution (200-1000 bp) with a peak size of approximately 300 bp for the prepared RNA-seq library samples. RNA-seq libraries constructed from the small amount of total RNA samples extracted from the mouse airway brushing samples were of high quality for the next step in the RNA-seq experiment.

Finally, the sequencing of these RNA-seq library samples was performed using the HiSeq 2500 platforms (Illumina, San Diego, CA). The reads generated were single-end (meaning the sequencing was performed for only one end of the molecular fragments of the library instead of sequencing both ends) with 50 nucleotides in length. The qualities of the RNA-seq reads were analyzed using the FastQC program (http://www.bioinformatics.babraham.ac.uk/projects/fastqc/). We multiplexed samples in a way that covered a sequencing depth ranging from 15 million to 32 million reads per RNA-seq sample. The quality scores of more than 96 of the bases of each sample were above 30, averaging around 40, greatly exceeding the threshold of 20.

In summary, the step-wise quality control procedures showed that the mouse airway brushing samples provided high quality RNA samples for subsequent RNA-seq experiments.

### Airway brushing samples suggested PI3K pathway is an early event during lung SCC development

**Figure 3:**
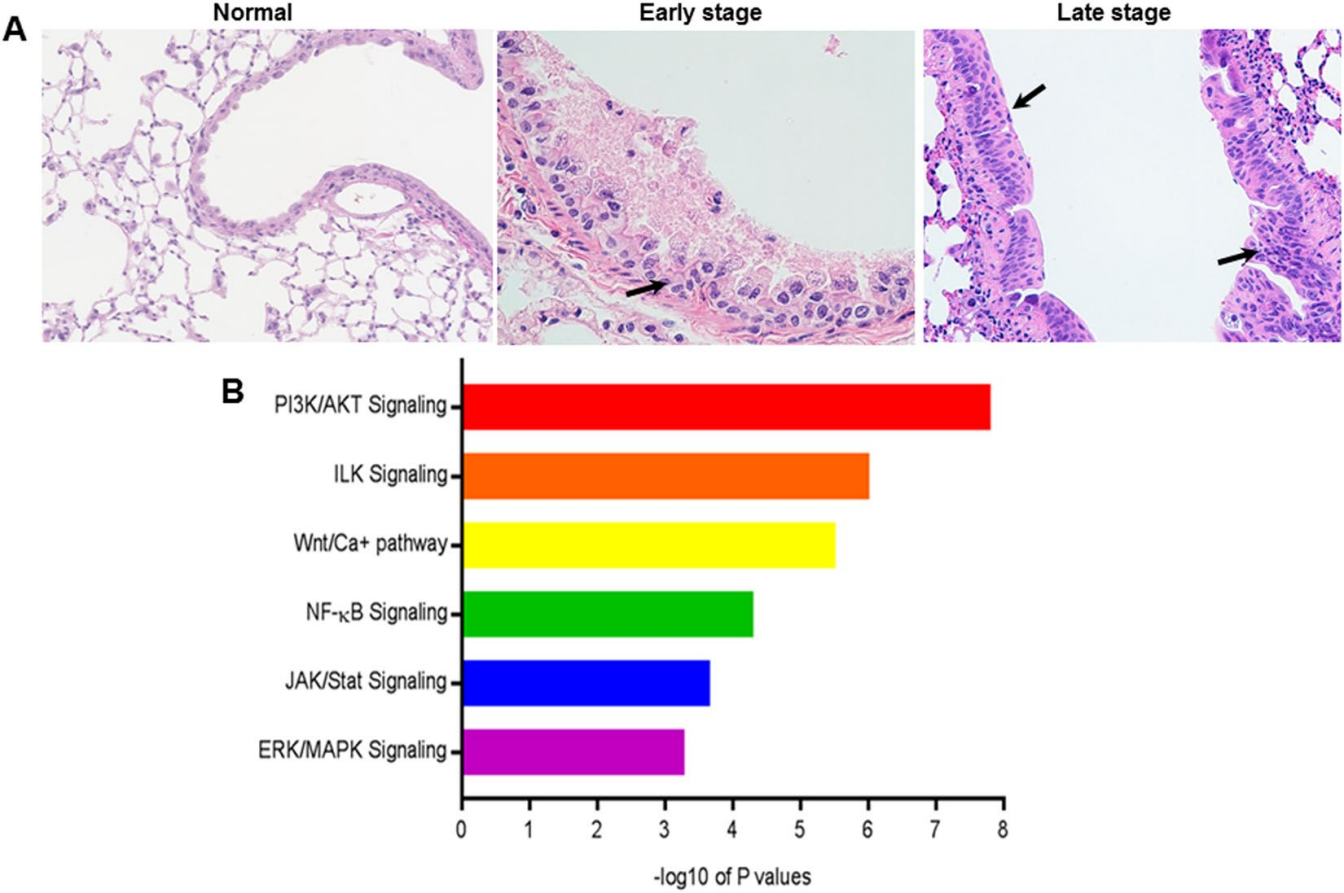
IPA analysis of brush samples of mouse lung SCC models vs normal mice. Significantly upregulated oncogenic pathways in the airway samples taken from mouse lung SCC models compared to the samples from the bronchial airways of normal control mice.

**Figure 4:**
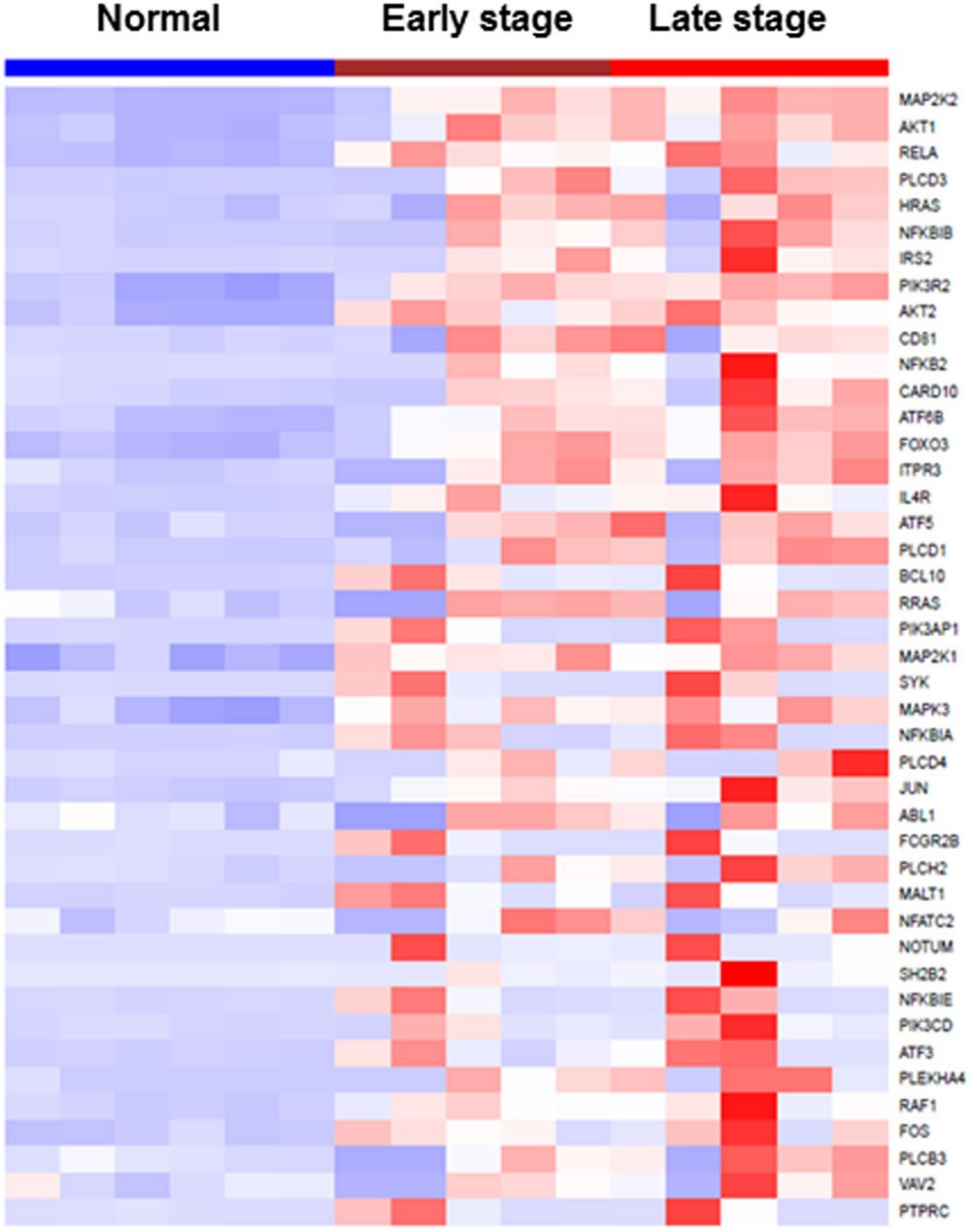
Heatmap of brush samples of mice lung SCC models vs normal mice. For the most significantly upregulated oncogenic pathway – PI3K/AKT signaling, a large number of PI3K/AKT pathway genes were significantly upregulated in the bronchial airways of lung SCC mice compared to the normal control mice.

The pre-processed sample RNA-seq reads were aligned to the mm9 mouse genome [UCSC version, July 2007) using Bowtie-TopHat (version 2.0.4, segment length 29 nt, 1 mismatch in segment permitted, for maximum sensitivity, coverage search performed^10,11^]. Read counts were obtained using HTSeq^12^. Batch effects were adjusted using the R package - RUVSeq^13^. Data normalization and differential expression analysis were performed using the statistical algorithms implemented in the statistical R packages - edgeR and limma^14,15^. FDR (False discovery rate) corrected *p*-values of less than 0.05 were used as criteria for significantly regulated genes. We also applied IPA analysis to the brush samples of lung SCC models and found that a set of oncogenic pathways were significantly upregulated in the bronchial airways of lung SCC mice compared to the airway samples taken from healthy control. As shown in our previous publication^16,17^, upon NTCU treatment, at early stage, the single layer bronchial epithelial cells are replaced with multiple layers of cells (hyperplasia) with increased production of keratin (Fig. 3A Early stage); while at late stage, lung SCC developed with large, flattened, and stratified cells with intra-cytoplasmic keratin/keratin pearls (Fig. 3A Late stage, arrow on the left) and/or atypical cells (irregular shape, increased nucleus/cytoplasm ratio, and so forth) with mitosis and loss of orderly differentiation replaced the entire thickened epithelium (Fig. 3A Late stage, arrow on the right). The most significantly upregulated pathways included: PI3K/AKT signaling, ILK signaling, Wnt/Ca^+^ signaling, NFƙB signaling, JAK/Stat signaling, and ERK/MAPK signaling (Fig. 3B). PI3K/AKT signaling was the most significantly upregulated oncogene pathway in the bronchial airway samples (P = 1.5×10^−8^, Fig. 3B) in the mouse lung SCC models. Heatmap analysis showed that an enriched set of PI3K/AKT genes were significantly upregulated in the bronchial airway samples of either the early (at risk) or the late stage (early lesion) mice with lung SCC relative to the control mice without any carcinogen exposure and lung SCC tumors (Fig. 4).

**Figure 5:**
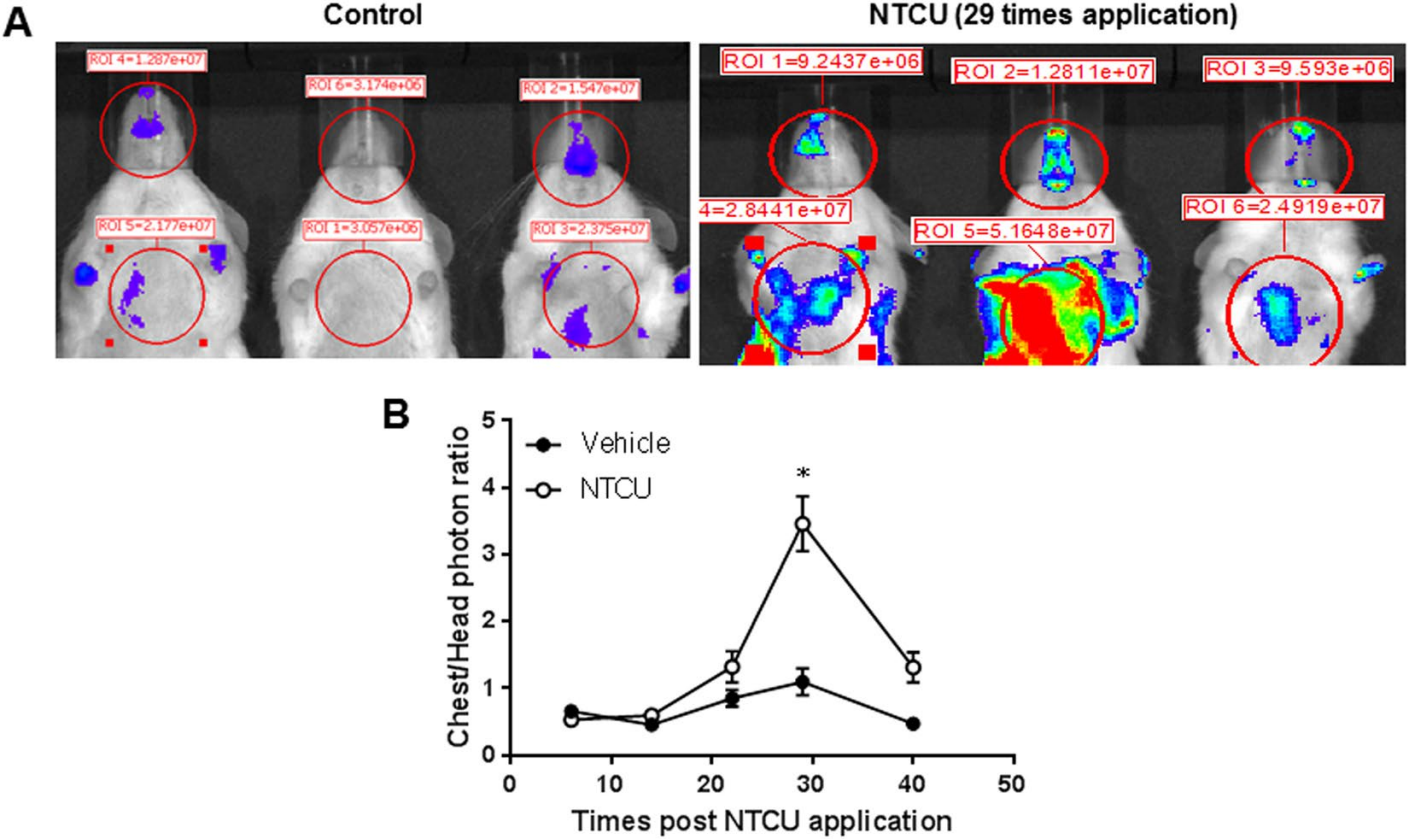
NTCU induced NFƙB activation along time. (**A**) representative live imaging of mice post NTCU topical application NFƙB-luc reporter mice. (**B**) Quantity data showing lung bioluminescence in NFƙB-luc reporter mice (normalized to head bioluminescence to correct for baseline differences) (*n* = 6 per group; *P = 0.05 compared with control group).

**Figure 6:**
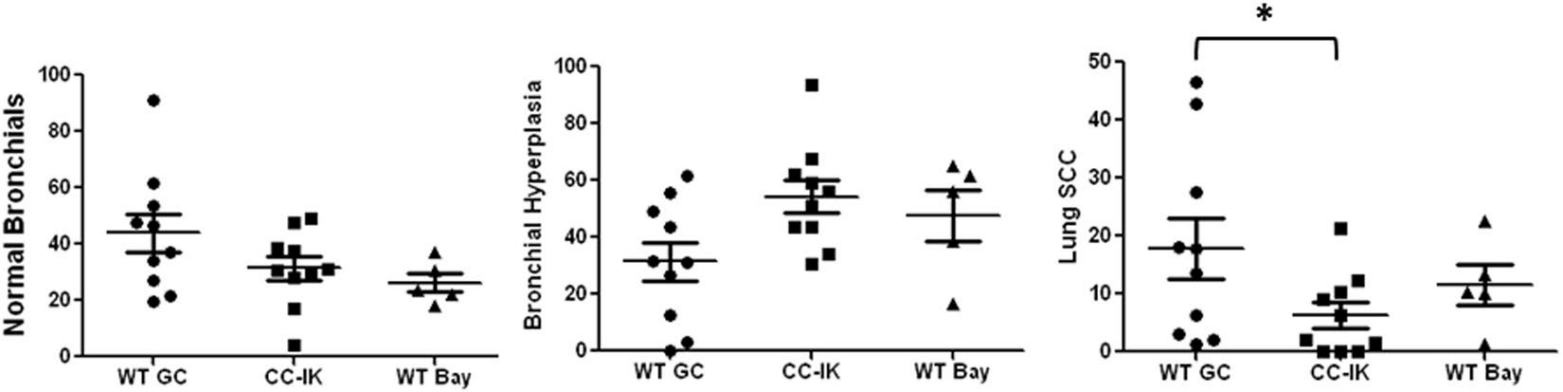
Inhibition of NFƙB in airway epithelium reduces NTCU-induced lung squamous cell carcinogenesis.

### NFƙB is activated during NTCU-induced lung SCC development

To verify whether NFƙB is indeed activated during NTCU-induced lung SCC development, we used NFƙB reporter mice that express luciferase under control of the NFƙB promoter. Mice were treated with NTCU twice per week and were imaged at baseline (before NTCU treatment) and weekly after each NTCU treatment. Bioluminescence showed that NFƙB activity was induced in these reporter mice by week 14 (Fig. 5, NFƙB activity reaches its peak by week 30) and then slowly returned to baseline (Fig. 5), suggesting that NFƙB activation is an early event during lung SCC development.

**Figure 7:**
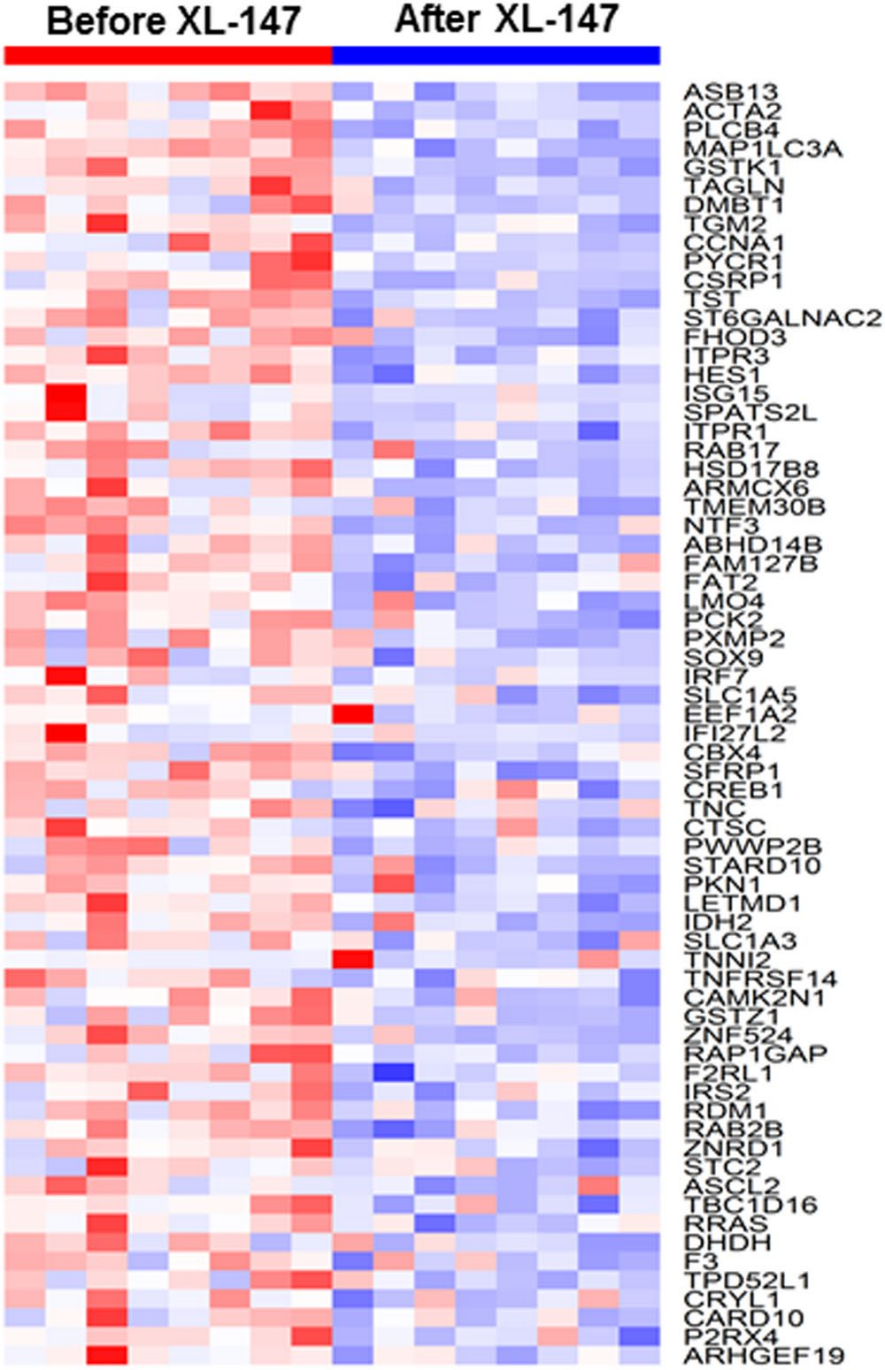
Heatmap of brush samples of mice lung SCC models before and after XL-147 treatment. The PI3K/NFκB pathway activation upregulated gene expression signature was significantly downregulated after XL-147 treatment.

### Blocking PI3K/NFƙB pathway with dominant negative IƙBα super-repressor or small molecule inhibitor prevents lung SCC

Based on the identification of NFƙB activation in airway epithelial cells after NTCU exposure, we used inducible transgenic mice that selectively express a dominant negative form of IƙBα-DN (super-repressor) under the control of the Clara cell-specific CC10 promoter^18^. After addition of doxycycline (dox), expression of IƙBα-DN in the lungs of these transgenic mice has been shown to block NFƙB activation^18^. To investigate the impact of NFƙB pathway on the development of lung squamous cell carcinoma, we bred IƙBα-DN (Swiss background over nine generations) with CCSP-rtTA mice (A/J background) mice. The F1 AJ-Swiss CCSP-IκƙBα-DN (CC-IK) mice were treated with dox for 1 week, followed by twice per week NTCU skin painting that continued for 26 weeks, wildtype littermates with same dox treatment were used as control. Lungs were harvested after 29 weeks. NTCU induced lung SCC in all control mice, the distributions of lesions were analyzed as we previously published^17,19^ . Briefly, after serial H&E staining, phenotype of each bronchus was evaluated, and distribution of each phenotype (normal, hyperplasia, metaplasia, carcinoma *in situ*, and SCC) was then calculated as the percentage to total bronchus analyzed for each mouse. Near 20% of bronchus from control mice showed sign of lung SCC (Fig. 6), however, only 6% of total bronchus from CC-IK mice developed lung SCC phenotype (Fig. 6), with several mice showed no sign of SCC at all. We also tested a specific NFƙB inhibitor Bay-11082 (Fig. 6) and a PI3K-specific inhibitor XL-147 (data not shown) in this NTCU lung SCC model; both showed intermediate inhibition on lung SCC development, suggesting PI3K/NFƙB pathway could be a novel target to effectively prevent lung SCC.

### RNA-seq experiment showed the reversal of PI3K/NFƙB pathway after inhibitor treatment

We applied the airway brushing technique to NTCU-induced lung SCC mice treated with the PI3K specific inhibitor XL-147. Bronchial airway brush samples were obtained from eight mice before and after XL-147 treatment. We performed total RNA extraction and subsequent RNA-seq of the paired airway samples from the eight mice before and after XL-147 treatment. The RNA-seq data analyses procedures were conducted in the same manner as mentioned in the previous paragraphs. In addition, we compared our results with the archived PI3K/NFƙB activation gene expression signatures from recent studies^5,20^. We found that PI3K/NFƙB pathway activation upregulated a gene expression signature which was significantly downregulated by XL-147 treatment (Fig. 7). This was provided clear evidence of the anti-PI3K pathway activity effect mediated by XL-147 administration. It also revealed that the PI3K/NFƙB pathway inhibition by XL-147 treatment can be detected in the bronchial airway of lung SCC mice, demonstrating the effectiveness of utilizing airway brushing techniques in detecting altered molecular events like gene expression changes that contribute to the efficacy of chemopreventive agents.

### Validation of RNA-seq results using independent data sets and the secondary approach of target RNA-seq

The amounts of the total RNA samples that were obtained by the trachea-bronchus brushing method were very little. Most of the RNA samples had been used up in the initial RNA-seq library construction. Therefore, we utilized additional sets of mice airway samples subjected to targeted RNA-seq to validate the initially identified gene set. Targeted RNA-seq allowed us to study the large set of genes to be validated using very limited mice airway samples and significantly improved the sequence coverage of transcripts of interest that may be present in low amounts, thus saving costs and simplifying analysis. The new mice data set (named “ValidationSet1”) to validate the PI3K/AKT gene set (Fig. 4) consisted of airway samples from 6 normal mice, 10 early-stage SCC mice and 10 late-stage SCC mice. The new mice data set (named “ValidationSet2”) to validate the PI3K/NFƙB gene set (Fig. 7) consisted of airway samples from 6 before and 6 after XL-147 treatment mice.

On the basis of the new data set - ValidationSet1 generated by the secondary method, 29 of 43 upregulated genes in the PI3K/AKT gene set shown in Fig. 4 had been validated (Fig. S3). ValidationSet2 verified 48 out of 68 genes in the PI3K/NFƙB gene set down-regulated by XL-147 as shown in Fig. 7 had been similarly down-regulated (Fig. S4). Therefore, the majority of the genes in the key PI3k gene sets as initially identified by RNA-seq were validated.

## Discussion

The airway brushing techniques described in this protocol are not restricted to tumor-related studies or RNA-seqspecific applications. Exposure to ambient air pollution is a leading risk factor for global disease burden, and the resulting chronic lung diseases such as lung cancer and chronic obstructive pulmonary disease (COPD) are among the top five leading causes of death. Chronic epithelial injury and aberrant repair processes are believed to be the major cause of these diseases. However, it is still unclear whether the primary lesions occur in the airways, the alveoli, or both epithelial compartments. Although the alveoli execute the main function of the lungs, multiple research studies indicate that the primary pathogenic abnormalities may occur in the airways^21–25^. Persistent Bronchial dysplasia (BD) is associated with the development of invasive SCC in humans^25^. Similarly, in mouse lung SCC model induced by NTCU, lung epithelial dysplasia was reported to start in the distinct airway locations, tracheal epithelium, and then followed by basal cell metaplasia of the bronchial epithelium^24^.

As genetically identical mice (inbred mice) of the same age that are housed under the same conditions possess significant amount of variation in gene expression, this approach introduces a background of genetic noise that can limit the ability to detect differences in gene expression resulting from pathological processes. Different hormonal milieu, different state of the immune system, and the degree of inflammatory activity may all attribute to these genetic variations^26^. In addition, the process of killing the animal may itself cause global changes in gene expression that are inconsistent from one mouse to the next, which is particularly problematic in studies focusing on stress-responsive genes.

Therefore, our current airway brushing protocol provided a non-lethal biopsy method for rodents that allow sampling at multiple time points without the need to euthanize the animals. The current protocol describes the method of collecting airway epithelial cells, primarily from the upper airways. For airway epithelial cells in the bronchus or close to the distal region, a modified lobectomy (Fig. S5) from a previous publication can be used^27^, which enables us to collect lung tumors or one of the lung lobes without euthanizing mouse and is mimicking pre-surgical approach clinically.

In the study, we compared homemade brush (Sanded PE-10 tube) with commercially available brush (Conmed cytological brush), we found that even though homemade brush was able to get enough air way epi cells, however, majority was with damaged cell membrane structure, which will fail the morphological analysis of the epithelial cells, furthermore, leads to the biological damage to the liberated components and their content, such as DNA, RNA etc., compromise subsequent analysis. Commercially available brush on the other hand, got mostly intact epithelial cells, which allows for their use for primary tissue culture, further lineage analysis with immunohistochemistry, or immunofluorescence staining; or RNA, DNA, or protein extraction for further transcriptional, genomic, or proteomic analyses to identify critical pathways or factors associated with disease progression or treatment.Identifying the transcriptome changes in bronchial airway epithelial cells that are able to detect the risk for lung cancer as well as the therapeutic effects of chemoprevention agents remain a novel and appealing subject of research. By performing RNA-seq of the airway samples from the lung SCC mouse model that were induced by the carcinogen - NTCU, we demonstrated that the PI3K/AKT/NFƙB pathways were activated in the normal appearing bronchial airway epithelial cells of NTCU mice, which can be reversed by treatment with XL-147 (a PI3K Inhibitor). This agrees with our previous findings using using small-animal imaging techniques (MRI and CT) that early stage SCCs are centrally located along the trachea^17^, while diffuse tumor tissue fills the lungs in the later stages of the disease.

Our results indicated that airway gene expression studies may serve as an alternative tissue biomarker for alterations that occur in the peripheral cells of the lung lower airways. These may have significant clinical implications for the development of better and noninvasive approaches for the prognosis of lung SCC and monitoring the treatment effects on this type of cancer.

## Electronic supplementary material

Supplementary Information
