# Supplementary material for: Airway brushing as a new experimental methodology to detect airway gene expression signatures in mouse lung squamous cell carcinoma
Source: Sci Rep. 2018 Jun 11;8:8895. doi: 10.1038/s41598-018-26902-7 (PMC5995924; doi:10.1038/s41598-018-26902-7)

Airway brushing as a new experimental methodology to detect airway gene expression signatures in mouse lung squamous cell carcinoma

Jing Pan[1,2,#], Donghai Xiong[1,2,,#], Qi Zhang[1,2,#], Eva Szabo[3], Mark Steven Miller[3], Ronald A. Lubet[3], Yian Wang[1,2]and Ming You[1,2]

[1]Cancer Center,Medical College of Wisconsin, 8701 Watertown Plank Road, Milwaukee, WI 53226, USA;
[2]Department of Pharmacology & Toxicology, Medical College of Wisconsin, 8701 Watertown Plank Road, Milwaukee, WI 53226, USA;
[3]Chemopreventive Agent Development Research Group, Division of Cancer Prevention, National Cancer Institute, 9609 Medical Center Drive, Rockville, MD 20850.

#These authors contributed equally to this work.

Correspondence to: Ming You,MD, PhD, Cancer Center and Department of Pharmacology & Toxicology, Medical College of Wisconsin, 8701 Watertown Plank Road, Milwaukee, WI 53226, USA (e-mail: myou@mcw.edu).

Mice populations and bronchial brush sample collection

For comparison, we set up a negative control group of Swiss mice that were not subjected to any treatment (called 'Normal' group). In parallel, the positive control group (only treated with carcinogen) and preventive agents treatment groups (carcinogene plus drug treatment) of mice were all treated with the carcinogen - NTCU at the same dose and duration of time (treated biweekly with topical 40 mmol/L NTCU for 2 weeks for "Early stage" model, and 20 weeks for "Late stage" model). For sample collection, we did bronchial brushing twice, one at 2 or 20 weeks before preventive agent intervention, and another at 6 or 26 weeks after preventive agent intervention.

RNA processing and RNA-seq experimentation.

Total RNA samples were extracted from the airway brush samples using a Qiagen RNeasy Mini Kit according to the manufacturer's instructions. The quality of the total RNA samples obtained was high, with RNA integrity number values in the range of 9–10. We used the TruSeq RNA Library Preparation Kit v2 to construct the RNA-seq libraries. The sequencing of these RNA-seq library samples was performed by the Medical College of Wisconsin Human and Molecular Genetics Center Sequencing Core using the HiSeq 2500 platforms (Illumina). The reads generated were single end and 50 nucleotides in length. The qualities of the RNA-seq reads were analyzed using the FastQC program (http://www.bioinformatics.babraham.ac.uk/projects/fastqc/). The coverages ranged from 15 million to 32 million reads per RNA-seq sample. The quality scores of >95.3% of all the bases of each sample are >30, averaging around 40, greatly exceeding the threshold of 20.

RNA-seq read alignment and differential expression analysis.

The preprocessed sample RNA-seq reads were aligned to the mm9 mouse genome (UCSC version, July 2007) using Bowtie-TopHat (version 2.0.4, segment length 29-nt, 1 mismatch in segment permitted for maximum sensitivity, coverage search performed). Read counts were obtained using HTseq{Anders, 2015 #19}. Batch effects were adjusted using the R package RUVSeq{Risso, 2014 #20}. Data normalization and differential expression analysis were performed using the statistical algorithms implemented in the statistical R package edgeR{Robinson, 2010 #22;Ritchie, 2015 #21}. FDR-corrected P values of less than 0.05 were used as criteria for significantly regulated genes. The list of differentially expressed genes in the bronchial airway samples between early or late stage lung SCC mice and non-lesion control mice as well as between before and after XL-147 treatment were analyzed by IPA (http://www.ingenuity.com). The two major modules in IPA, i.e., 'Canonical Pathways' and 'Networks', were adopted for pathway and network enrichment analysis. The

heatmaps of gene expression were generated using the R package – heatmap3 (https://cran.r-project.org/web/packages/heatmap3/).

Figure Legends:

Figure S1. RNA Integrity of brushed samples. RNA extracted from brushed samples was checked using the Agilent RNA 6000 Pico Chip analysis. The RIN value of the two test samples was greater than 8, indicating very high quality RNA samples**.**

Figure S2. RNA-sequencing library quality check from cells collected by tracheal-bronchus brushing. Agilent High Sensitivity DNA Chip analysis of the prepared RNA-sequencing libraries based on the total RNA samples extracted from mouse lung epithelial cells collected by tracheal-bronchus brushing.

Figure S3. ValidationSet1 generated by the target RNA-seq method showed that 29 of 43 genes in the PI3K/AKT gene set shown in Figure 4 had been validated. The multiple testing adjusted p values for the t-tests comparing "Early_stage" samples to "Normal" samples or "Late_stage" samples to "Normal" samples were all less than 0.05.

Figure S4. ValidationSet2 verified that 48 of 68 genes in the PI3K/NF-□B gene set down-regulated by XL-147 treatment as shown in Figure 7 had been similarly down-regulated. The multiple testing adjusted p values for the t-tests comparing "After XL-147" samples to "Before XL-147" samples were all less than 0.05.

Figure S5. Lobectomy surgery protocol for extracting lung tumors or lung lobe from live mouse. A. B. Prepare surgical site, the left thoracic wall is shaved and disinfected; after anesthesia, mouse is intubated and mechanically ventilated; **C.** An incision is performed in the second intercostal space and extended to the pleural cavity; **D.** The chest is kept open with two retractors;**E.** the left lung lobe is lifted gently through the opening; **F.G.** Tumors on this lobe are then extracted with scissor, and flash frozen in liquid nitrogen; **H.** The muscle and subcutaneous tissues of the thorax are closed with 7-0 sutures, and finally skin is then closed with 7-0 sutures. Mechanical ventilation is weaned at the onset of spontaneous breathing, and mice are monitored in ICU until ambulatory.

Fig S1.

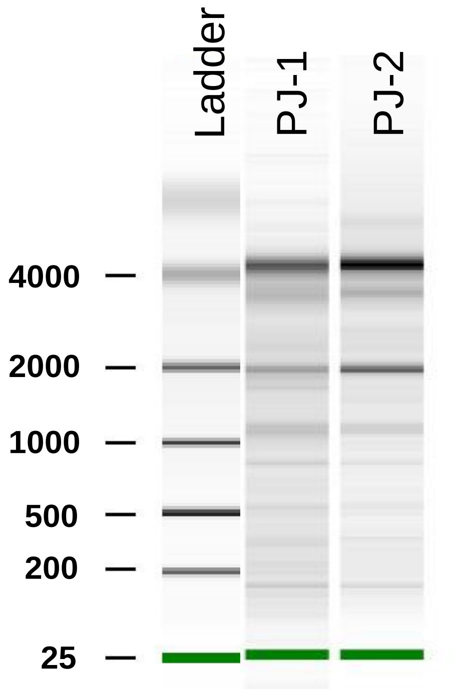

Fig S2.

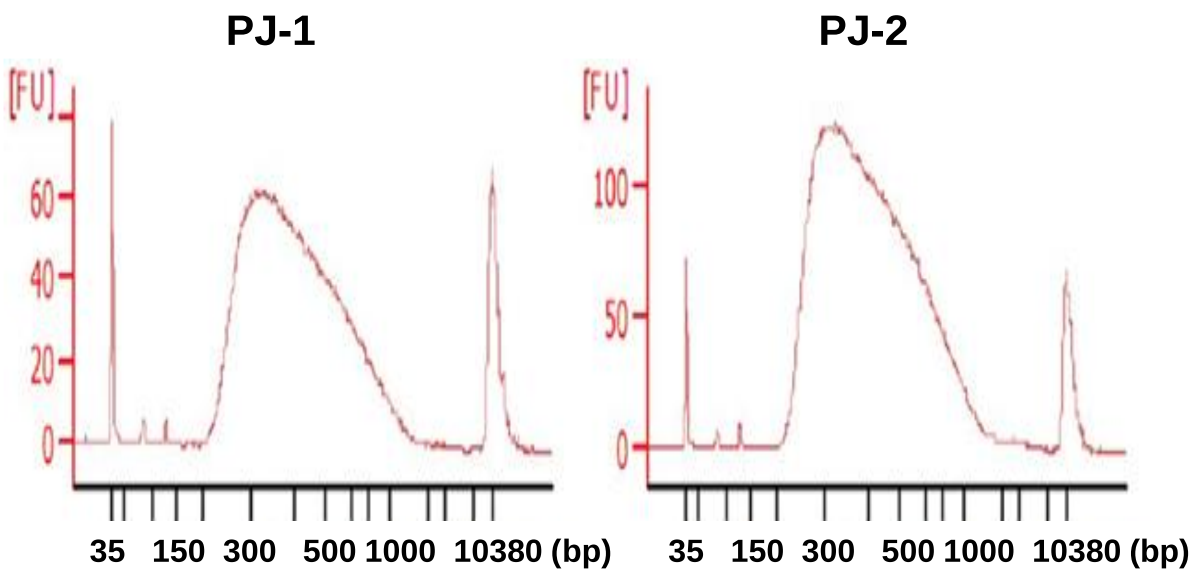
PJ-1
[FU]
60
40
20
0
35 150 300 500 1000 10380 (bp)
PJ-2
[FU]
100
50
0
35 150 300 500 1000 10380 (bp)

Fig S3.

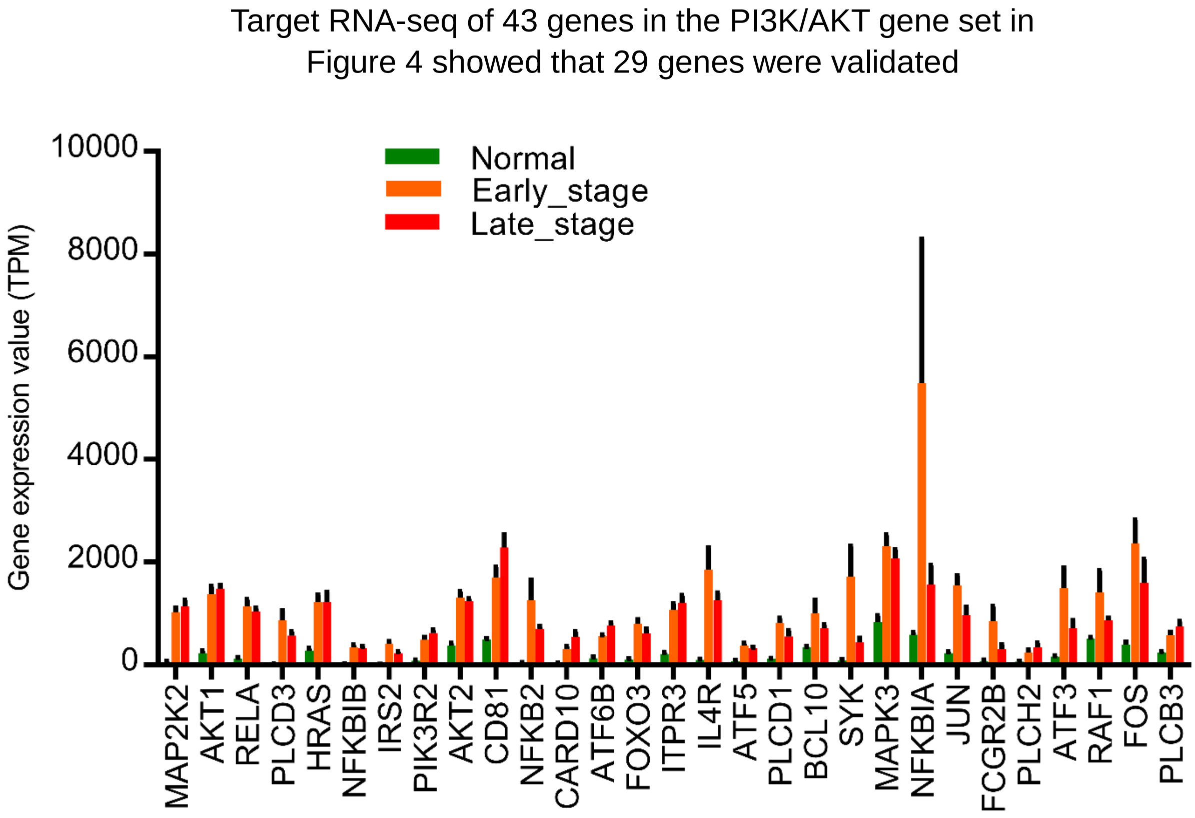
Target RNA-seq of 43 genes in the PI3K/AKT gene set in
Figure 4 showed that 29 genes were validated
Normal
Early_stage
Late_stage
Gene expression value (TPM)
10000
8000
6000
4000
2000
0
MAP2K2
AKT1
RELA
PLCD3
HRAS
NFKBIB
IRS2
PIK3R2
AKT2
CD81
NFKB2
CARD10
ATF6B
FOXO3
ITPR3
IL4R
ATF5
PLCD1
BCL10
SYK
MAPK3
NFKBIA
JUN
FCGR2B
PLCH2
ATF3
RAF1
FOS
PLCB3

Fig S4.

Target RNA-seq of 68 genes in the PI3K/ NF-κB gene set in Figure 7 showed that 48 genes were similarly down-regulated by XL-147 treatment

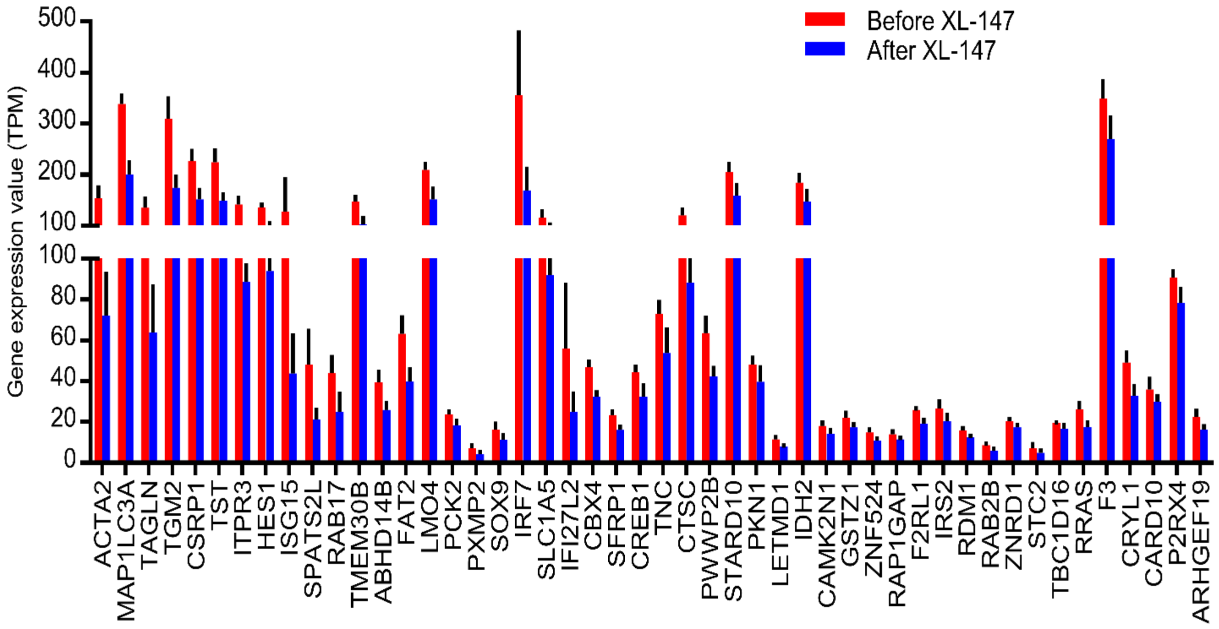

Fig S5.

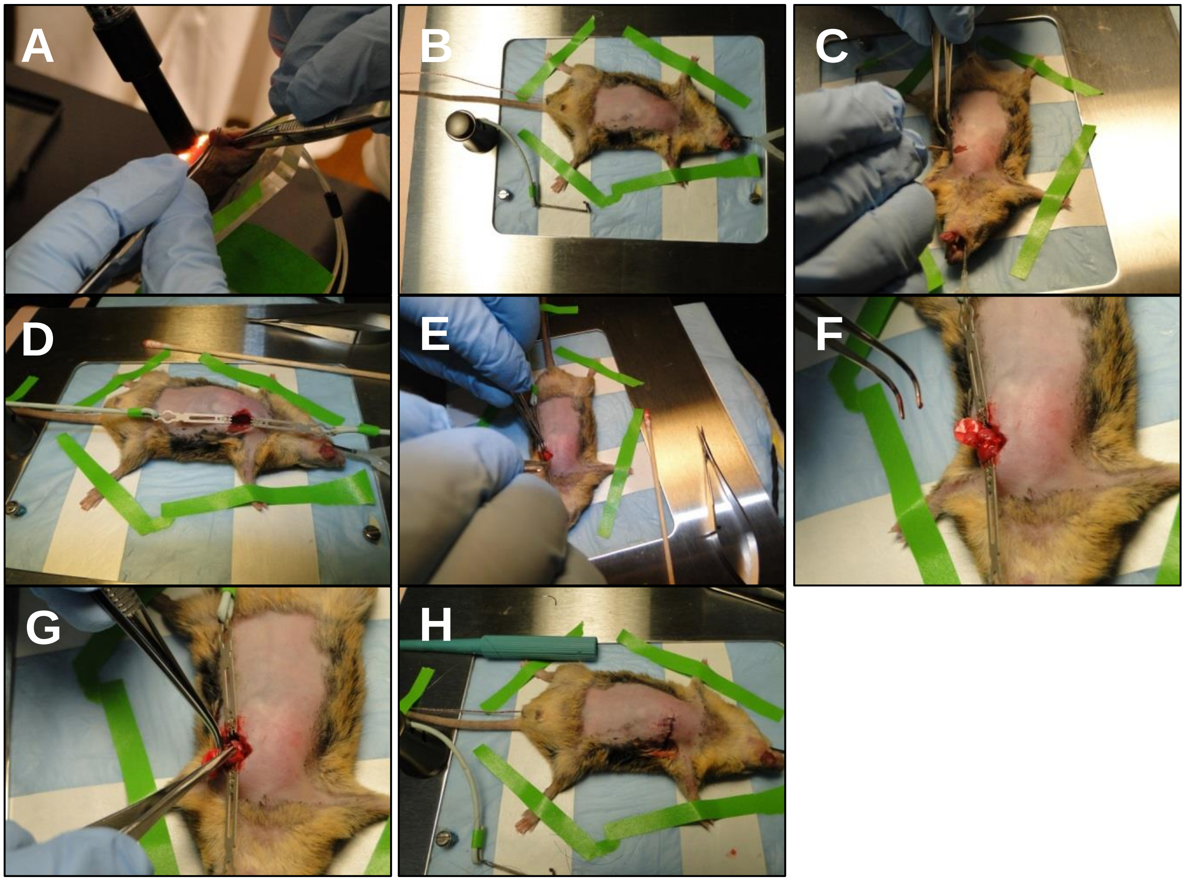

Supplement: Supplementary file 1 — Supplementary Information [file 41598_2018_26902_MOESM1_ESM.pdf]
